# Anaerostipes stercorihominis sp. nov., isolated from human faeces

**DOI:** 10.1099/ijsem.0.007276

**Published:** 2026-09-07

**Authors:** Tomoki Tanabe, Atsushi Hisatomi, Moriya Ohkuma, Mitsuo Sakamoto, Akihito Endo

**Affiliations:** 1 Department of Nutritional Science and Food Safety, Faculty of Applied Bioscience, Tokyo University of Agriculture, Setagaya, Tokyo 156-8502, Japan; 2 Microbe Division/Japan Collection of Microorganisms, RIKEN BioResource Research Center, Tsukuba, Ibaraki 305-0074, Japan; 3 NODAI Culture Collection Center, Tokyo NODAI Research Institute, Tokyo University of Agriculture, Setagaya, Tokyo 156-8502, Japan

**Keywords:** *Anaerostipes stercorihominis*, *Anaerostipes faecis*, *Anaerostipes hominis*, butyrate production, lactate metabolism, taxonomy

## Abstract

The genus *Anaerostipes* is one of the major butyrate-producing genera in the gut microbiota of healthy human adults. During a culturomics study of the human gut, strain 17EGHSPOS18-1^T^ was isolated from the faeces of a healthy adult. Here, we conducted a taxonomic study of the strain. Phylogenetic analysis based on 16S rRNA gene sequences placed strain 17EGHSPOS18-1ᵀ in the *Anaerostipes* cluster. This placement was supported by a core gene-based phylogenetic analysis. Strain 17EGHSPOS18-1^T^ shared average nucleotide identity (ANI) values of <80% and digital DNA–DNA hybridization (dDDH) values of <30% with known *Anaerostipes* spp., indicating that it does not belong to any known species of the genus *Anaerostipes*. Physiological and chemotaxonomic characteristics, including carbohydrate utilization patterns, enzyme activities, major cellular fatty acid profiles and major fermentation end-product profiles, further distinguished the strain from the known *Anaerostipes* spp. The novel strain was able to metabolize only a limited number of carbohydrates, consistent with its restricted repertoire of glycoside hydrolase family proteins. These findings indicate that 17EGHSPOS18-1^T^ represents a novel species, for which the name *Anaerostipes stercorihominis* sp. nov. is proposed. The type strain is 17EGHSPOS18-1^T^ (= JCM 38261^T^ = DSM 121506^T^). Furthermore, during this taxonomic study, we found that *Anaerostipes hominis* and *Anaerostipes faecis* share ANI and dDDH values above the species discrimination thresholds, indicating that they should be assigned to the same taxon. Based on the data, we propose *A. faecis* as a later heterotypic synonym of *A. hominis*.

## Introduction

The human gut harbours a tremendous number of microbial cells with vast diversity, which play important roles in maintaining host health through metabolic, immunological and physiological interactions. Among the diverse microbial groups in the gut, members of the family *Lachnospiraceae* are recognized as major producers of short-chain fatty acids (SCFAs), particularly butyrate, which contributes to intestinal homeostasis and systemic health [1, 2].

The genus *Anaerostipes*, belonging to the family *Lachnospiraceae*, was established by Schwiertz *et al*. with the description of *Anaerostipes caccae* in 2002 [3]. A notable feature of the genus is its ability to produce butyrate as a major end product not only from carbohydrates but also from lactate in combination with acetate [4, 5]. At the time of writing (May 2026), eight validated species, one yet-to-be validated species and three *Candidatus* species have been described within the genus *Anaerostipes* based on the List of Prokaryotic names with Standing in Nomenclature (https://lpsn.dsmz.de/species/anaerostipes-coli). All members have been isolated from animal gut, including humans, chickens, pigs and mice [3, 6–8]. The depletion of specific *Anaerostipes* spp. has been inversely correlated with human health status, and members of this genus are thus regarded as potential candidates for live biotherapeutic products targeting several disorders [9, 10]. Accordingly, the metabolic properties of prebiotic oligosaccharides have been characterized in *Anaerostipes* spp. to promote their growth and enhance butyrate production [11].

During a culturomics study of the human gut microbiota, a single strain potentially belonging to the genus *Anaerostipes* was isolated from the faeces of a healthy adult. The present study taxonomically characterized the strain and proposes it as a novel species in the genus *Anaerostipes*. Furthermore, our taxonomic study revealed that *Anaerostipes hominis* and *Anaerostipes faecis* belong to the same taxon and should be reclassified accordingly. The present study contributes to the refinement of the taxonomy of *Anaerostipes*, an important butyrate-producing genus in the human gut.

## Bacterial strains and growth conditions

This study was approved by the RIKEN Ethics Committee (approval no. Tsukuba 27-1). Faecal samples were obtained from two healthy Japanese volunteers (subject no. 17, a 30-year-old man and subject no. 18, a 26-year-old woman) in April 2022. Written informed consent was obtained from the volunteers.

To isolate ethanol-resistant gut bacteria, 0.1 g of faecal sample was suspended in 1 ml of 70% (v/v) ethanol and placed under aerobic conditions for 5 h. Following the ethanol treatment, the suspension was centrifuged and the resulting pellet was resuspended in 1 ml of faecal culture supernatant. This supernatant was prepared by culturing a 10-fold dilution of a faecal sample in a yeast casitone fatty acids (YCFA) broth [Japan Collection of Microorganisms (JCM) medium no. 1130, https://www.jcm.riken.jp/cgi-bin/jcm/jcm_grmd?GRMD=1130] at 37 °C under anaerobic conditions for 2 days. Specifically, the pellet obtained from subject no. 17 was resuspended in the supernatant derived from a culture of subject no. 18. The resulting suspension was serially diluted in pre-reduced PBS and plated onto Eggerth–Gagnon agar (JCM medium no. 14, https://www.jcm.riken.jp/cgi-bin/jcm/jcm_grmd?GRMD=14&MD_NAME=). Plates were incubated at 37 °C for 1 day under anaerobic conditions (H_2_:CO_2_:N_2_=5:5:90) in an anaerobic chamber (model Bactron 300, Sheldon Manufacturing). Pre-reduced PBS was prepared by purging with anaerobic gas (CO_2_:N_2_=10:90) prior to autoclaving using an oxygen-removal unit. Strain 17EGHSPOS18-1^T^, originating from subject no. 17, was isolated during this process.

The following strains were included as reference strains for physiological and chemotaxonomic analyses: *Anaerostipes amylophilus* JCM 34801^T^, *Anaerostipes butyraticus* JCM 17466^T^, *A. caccae* JCM 13470^T^, *Anaerostipes faecalis* NBRC 114896^T^, *Anaerostipes hadrus* ATCC 29173^T^, *A. hominis* JCM 32275^T^, *Anaerostipes rhamnosivorans* DSM 26241^T^ and *Roseburia inulinivorans* JCM 17584^T^. JCM, NITE Biological Resource Center (NBRC) and American Type Culture Collection (ATCC) strains were obtained from the JCM, NBRC and ATCC, respectively. These reference strains and the strain 17EGHSPOS18-1^T^ were cultured in brain heart infusion (BHI, Oxoid) broth supplemented with 20 g l^−1^ yeast extract, 27 mM sodium acetate tri-hydrate, 0.5 g l^−1^ l-cysteine and 2 g l^−1^ d-fructose (BHIS) [12] at 37 °C for 24 h. The BHIS broth was purged with N_2_ gas before autoclaving.

## 16S rRNA gene phylogenies

The nearly complete 16S rRNA gene sequence of strain 17EGHSPOS18-1^T^ was determined, as described previously [13]. The 16S rRNA gene sequences of the major human gut butyrate producers in the family *Lachnospiraceae* including *Anaerostipes* spp. were retrieved from the National Center for Biotechnology Information (NCBI) database. Pairwise sequence similarities were calculated using blastn. Multiple sequence alignments were performed with the MUltiple Sequence Comparison by Log-Expectation, and a maximum-likelihood (ML) phylogenetic tree was constructed using SeaView (version 5) [14, 15]. Bootstrap analysis was performed with 100 replicates to estimate the confidence of tree topologies. The resulting trees were visualized and illustrated using iTOL [16].

In the 16S rRNA gene sequence analysis, strain 17EGHSPOS18-1^T^ showed the highest sequence similarity of 95.7% with *A. faecalis*, followed by 95.0% with *A. amylophilus* and 94.9% with *A. hadrus* (Table S1, available in the online Supplementary Material). The ML phylogenetic tree placed the strain within the *Anaerostipes* cluster, showing phylogenetic relatedness to *A. amylophilus*, *A. butyraticus*, *A. faecalis* and *A. hadrus* (Fig. S1). In the phylogenetic analysis, *A. hominis* and *A. faecis* shared high sequence similarities (99.6%) and formed a tight cluster with very short branches (Fig. S1 and Table S1), indicating a close phylogenetic relationship between the two species.

## Genome features

Genomic DNA was extracted from cells of 17EGHSPOS18-1^T^ as described previously [17] and used for draft genome sequencing on the Illumina platform. Reads were assembled using Platanus_B (version 1.1.0) with default settings, and sequences shorter than 300 bp were eliminated. Gene prediction and annotation of the resulting genome were performed using the DDBJ Fast Annotation and Submission Tool (DFAST) (version 1.2.4) (https://dfast.ddbj.nig.ac.jp/). The genome sequences of the type strains of *Anaerostipes* spp. were obtained from the NCBI database (Table S2). Average nucleotide identity (ANI) values were determined for all pairwise comparisons using blast+ using PyANI (version 0.2.12) [18]. Digital DNA–DNA hybridization (dDDH) values were calculated using the Genome-to-Genome Distance Calculator version 3.0 (http://ggdc.dsmz.de/distcalc2.php) [19]. A core gene phylogenetic tree was constructed using the Up-to-date-Bacterial Core Genes version 2 (UBCG2) set with default settings to assess genome-level phylogenetic relationships [20]. Metabolic pathway in each strain was predicted using Kyoto Encyclopedia of Genes and Genomes (KEGG) Automatic Annotation Server by assigning KEGG Orthology numbers to each predicted coding DNA sequences [21], and glycoside hydrolase (GH) family proteins were predicted using dbCAN3 in the carbohydrate-active enzymes database [22].

**Fig. 1.**
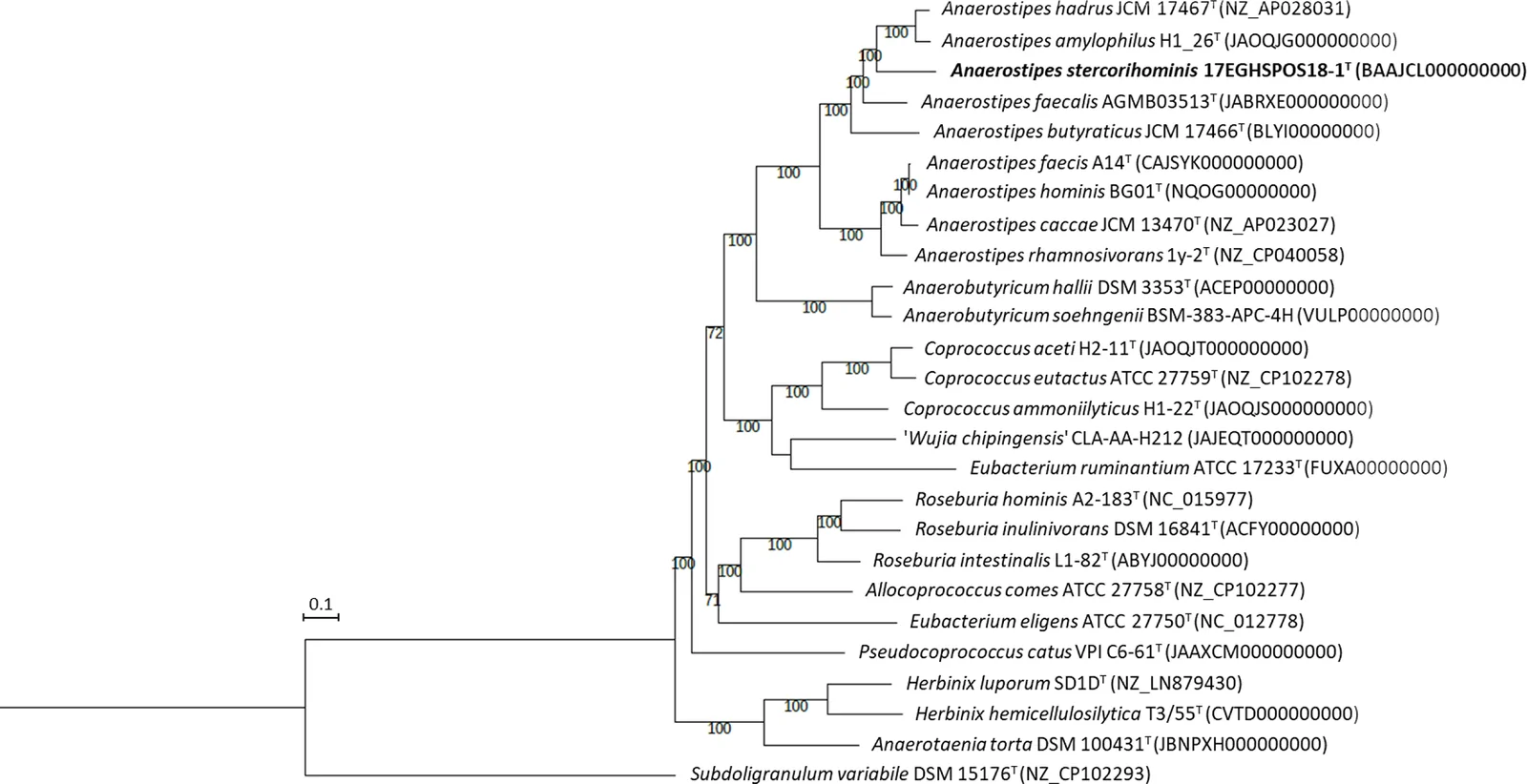
Phylogenomic tree generated using the UBCG2 set based on amino acid sequences, showing the relationship between *A. stercorihominis* sp. nov. and major gut butyrate-producing bacteria in the family *Lachnospiraceae*. Bootstrap values (>70%) based on 100 replicates are shown at the branch nodes. *Subdoligranulum variabile* DSM 15176^T^ was used as the outgroup. The scale bar represents 0.1 substitutions per nt positions.

The genome of strain 17EGHSPOS18-1^T^ was 2.7 Mbp in size, which falls within the range reported for *Anaerostipes* spp., from 2.5 Mbp in *A. amylophilus* to 3.6 Mbp in *A. caccae* and *A. rhamnosivorans* (Table S2). The G+C content of the novel strain was 40.5 mol%, also within the range (37.0–44.5 mol%). The strain shared ANI values of <80% and dDDH values of <30% with all recognized *Anaerostipes* spp. (Table 1), indicating that 17EGHSPOS18-1^T^ does not belong to any known species in the genus. In the core gene phylogenetic tree, the strain was positioned within the *Anaerostipes* cluster (Fig. 1), closely related to *A. amylophilus*, *A. butyraticus*, *A. faecalis* and *A. hadrus*, indicating that the novel strain is a member of the genus *Anaerostipes*.

**Table 1.** dDDH (top, bold type) and ANI (bottom, normal type) values (%) within the genus *Anaerostipes*

			1	2	3	4	5	6	7	8	9
1	*A. stercorihominis* sp. nov.	17EGHSPOS18-1^T^	–	**19.7**	**19.3**	**19.4**	**19.5**	**19.9**	**19.2**	**20.1**	**20.3**
2	*A. hadrus*	DSM 3319^T^	75.5	–	**36.1**	**19.9**	**20.4**	**21.1**	**20.5**	**21.1**	**20.2**
3	*A. amylophilus*	H1_26^T^	75.6	88.0	–	**19.8**	**19.7**	**20.2**	**18.7**	**20.3**	**18.8**
4	*A. butyraticus*	JCM 17466^T^	75.3	75.4	75.0	–	**19.5**	**21.4**	**20.9**	**22.7**	**20.7**
5	*A. faecalis*	AGMB 03513^T^	75.9	76.7	76.3	76.2	–	**20.2**	**19.1**	**21.4**	**19.2**
6	*A. caccae*	JCM 13470^T^	72.9	72.9	72.5	73.8	73.0	–	**48.5**	**25.0**	**48.3**
7	*A. hominis*	BG01^T^	73.1	72.8	72.6	73.7	72.9	92.2	–	**28.1**	**91.1**
8	*A. rhamnosivorans*	1y-2^T^	72.8	73.0	72.5	73.7	72.9	81.3	83.1	–	**27.5**
9	*A. faecis*	A14^T^	73.0	72.9	72.5	73.7	72.8	92.1	98.8	82.9	–

In the genome relatedness analyses, *A. hominis* and *A. faecis* shared ANI and dDDH values of 98.8 and 91.1%, respectively (Table 1). These values were well above the commonly accepted species discrimination thresholds of 95–96% for ANI and 70% for dDDH [23]. The results indicate that *A. hominis* and *A. faecis* represent the same species and should therefore be regarded as taxonomic synonyms. Due to the high genomic similarity, *A. hominis* and *A. faecis* clustered tightly, with very short branches, in the core gene phylogenetic tree (Fig. 1).

All nine strains studied, including the novel strain 17EGHSPOS18-1^T^, possessed genes encoding butyryl-CoA:acetate-CoA transferase but lacked genes encoding butyrate kinase, indicating that butyrate is likely produced through the butyryl-CoA:acetate-CoA pathway. They also possessed l-lactate dehydrogenase, acetate kinase and formate C-acetyltransferase, which are key enzymes for the production of L-lactate, acetate and formate, respectively, whereas methylmalonyl-CoA/ethylmalonyl-CoA epimerase, a key enzyme for propionate production, was not detected in any of the strains. Strain 17EGHSPOS18-1^T^ possessed only 16 genes encoding GH family proteins, including GH3, GH13, GH23, GH25, GH73, GH77 and GH109, a smaller number than those identified in known *Anaerostipes* species [11].

## Physiology and chemotaxonomy

The physiological and chemotaxonomic characteristics of 17EGHSPOS18-1^T^ were studied using the methods described by Sakamoto *et al*. [24]. *A. amylophilus* JCM 34801^T^, *A. butyraticus* JCM 17466^T^, *A. faecalis* NBRC 114896^T^ and *A. hadrus* ATCC 29173^T^ were included as phylogenetically related reference strains. Cell morphology was observed using scanning electron microscopy following the method described by Ang *et al*. [25]. Since certain *Anaerostipes* species are known to form spores, spore-forming ability of strain 17EGHSPOS18-1^T^ was evaluated using 24-h-old cultures grown in BHIS broth and modified Gifu anaerobic medium (mGAM; Nissui) broth, using the phase-contrast microscopy and ethanol-treatment analysis as described by Kadowaki *et al*. [26].

Among the tested 14 carbohydrates, all strains metabolized d-glucose, d-fructose, d-mannose and d-mannitol but did not metabolize raffinose. All strains grew at pH values ranging from 6 to 8.

Strain 17EGHSPOS18-1^T^ displayed distinct carbohydrate metabolism profiles compared with those of the phylogenetically related reference strains. Notably, the strain did not grow on d-galactose, kestose, lactose or nystose – substrates that were well utilized by most of the reference strains (Table 2). Strain 17EGHSPOS18-1^T^ grew between 30 and 45 °C, with optimal growth at 37 °C. It exhibited relatively poor enzymatic activity in API ZYM tests compared with other *Anaerostipes* spp. NaCl tolerance slightly distinguished the strain from the reference strains. Cells usually occurred singly or in pairs and measured 0.5×2.0–5.0 µm in size (Fig. S2). No spore formation was observed in strain 17EGHSPOS18-1^T^ under the tested conditions.

**Table 2.** Differential characteristics among the tested strains Strains: 1, *A. stercorihominis* 17EGHSPOS18-1^T^; 2, *A. hadrus* ATCC 29173^T^; 3, *A. amylophilus* JCM 34801^T^; 4, *A. butyraticus* JCM 17466^T^; and 5, *A. faecalis* NBRC 114896^T^.

			1	2	3	4	5
Acid production from							
	Cellobiose		−	+	−	+	−
	d-Fucose		−	w	w	−	−
	d-Galactose		−	+	+	w	+
	d-Gluconate		−	+	−	+	−
	*myo*-Inositol		+	w	−	−	−
	Kestose		−	+	+	+	+
	Lactose		−	w	+	−	+
	Maltose		w	+	+	w	w
	Nystose		−	+	w	+	+
	d-Ribose		+	w	+	w	+
	Sucrose		w	+	w	+	−
Temperature for growth (°C):			30–45	30–42	30–45	37–45	37–45
	Optimum		37	30–42	30–42	37–45	37–45
NaCl tolerance (%)			<2	<1	<2	<1	<2
Enzyme activity:							
	Alkaline phosphatase		w	+	+	−	+
	Esterase (C4)		−	w	w	+	w
	Esterase lipase (C8)		−	−	−	−	w
	Acid phosphatase		−	+	+	+	+
	Naphthol-AS-Bl-phosphohydrolase		w	w	w	+	+
	β-Galactosidase		−	+	−	−	−
	α-Glucosidase		−	+	+	+	−
Major cellular fatty acids (%):			C_12:0_ (36.0), C_18:0_ aldehyde (17.4), C_18:0_ DMA (32.4)	C_12:0_ (56.6), C_11:0_ DMA (13.6), C_18:0_ DMA (10.6)	C_12:0_ (26.4), C_18:0_ aldehyde (16.4), C_11:0_ DMA (10.8), C_18:0_ DMA (31.3)	C_12:0_ (47.5), C_18:0_ DMA (12.8)	C_12:0_ (23.1), C_18:0_ DMA (17.7), C_18:1_ ω9c DMA (10.4)
End products from d-glucose:			Blf	BLaf	LBf	LBf	BLf

For the end products from D-glucose, upper- and lower-case letters indicate major end products (>10 mM) and minor end products (≤10 mM), respectively.

−, negative; +, positive; A, acetate; B, butyrate; F, formate; L, lactate; w, weakly positive.

SCFA production from d-glucose was tested in BHIS broth. SCFAs accumulated were determined according to Sakamoto *et al*. [24]. Strain 17EGHSPOS18-1^T^ produced butyrate as the major end product, along with trace lactate and formate, in BHIS broth (Table 3). Acetate was consumed during growth. This end-product profile differed from those of the reference strains. For example, *A. amylophilus* and *A. butyraticus* mainly produced lactate, whereas *A. hadrus* accumulated acetate instead of consuming. No strains accumulated propionate.

**Table 3.** End products (mM) formed after culturing of *Anaerostipes* spp

	Species	Strain	Butyrate	Lactate	Formate	Acetate
1	*A. stercorihominis* sp. nov.	17EGHSPOS18-1^T^	30.0	9.5	3.4	−12.3
2	*A. hadrus*	ATCC 29173^T^	20.4	14.3	2.1	9.5
3	*A. amylophilus*	JCM 34801^T^	10.8	42.9	2.2	−10.3
4	*A. butyraticus*	JCM 17466^T^	11.9	18.3	2.2	−4.1
5	*A. faecalis*	NBRC 114896^T^	30.9	16.0	4.9	−9.7

Values indicated with ‘−’ mean reduction of the acids after culturing of strains tested.

**Fig. 2.**
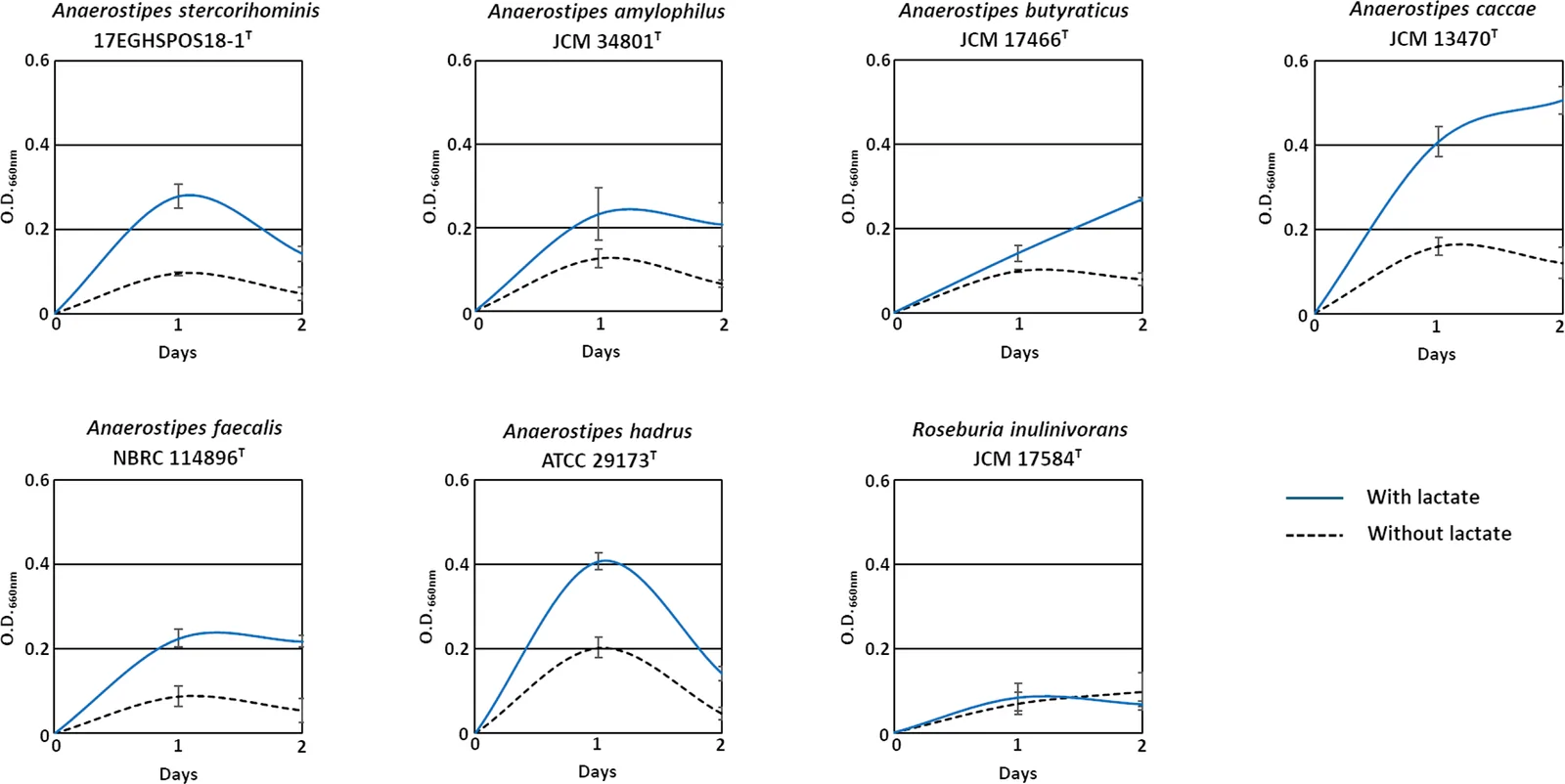
Growth of *Anaerostipes* spp. and *R. inulinivorans* in broth supplemented with or without lactate as the sole carbon source. Solid lines indicate growth in the presence of lactate, whereas dashed lines indicate growth in its absence.

To further study metabolic characteristics of the strain, its ability to grow on lactate – a typical characteristic in *Anaerostipes* species [4] – was tested. *A. caccae* JCM 13470^T^ was included as a positive control, whereas *R. inulinivorans* JCM 17584^T^ was used as a negative control here. A sugar-free YCFA broth supplemented with 35 mM dl-lactate [4] was used as the test medium, and sugar-free YCFA broth without lactate served as the control. The composition of YCFA broth was described elsewhere [27]. Growth was tested in triplicate and monitored spectrophotometrically at OD_660nm_. Growth of both the novel strain and reference strains was enhanced by the presence of lactate as the sole carbon source (Fig. 2), consistent with previous findings in *Anaerostipes* spp. [4], whereas growth of *R. inulinivorans* was not enhanced.

Fatty acid methyl esters were prepared from cells cultured in BHIS broth, and the cellular fatty acid composition was determined by the methods described previously [24, 28, 29]. The major cellular fatty acids of strain 17EGHSPOS18-1^T^ were C_12:0_, C_18:0_ dimethyl acetal (DMA) and C_18:0_ aldehyde (Table S3). This profile was similar to that of *A. amylophilus*, but differences in C_11:0_ DMA distinguished the novel strain from *A. amylophilus*.

For matrix-assisted laser desorption/ionization time-of-flight (MALDI-TOF) MS analysis, all strains were cultured on BHIS agar for 48 h. Colonies were processed using formic acid and acetonitrile following the manufacturer’s protocol. The spectra were generated with a MALDI-TOF MS Microflex LT system (Bruker Daltonics) according to the manufacturer’s recommended settings, and a dendrogram was created using the Biotyper compass explore software (Bruker Daltonics). Additional reference strains, *A. hominis* JCM 32275^T^ and *A. rhamnosivorans* DSM 26241^T^, were included in this analysis. The resulting dendrogram showed clustering patterns similar to those of the phylogenetic trees based on the 16S rRNA gene and core genes (Fig. S3). Strain 17EGHSPOS18-1^T^ produced a cluster with *A. amylophilus* JCM 34801^T^, *A. butyraticus* JCM 17466^T^, *A. faecalis* NBRC 114896^T^ and *A. hadrus* ATCC 29173^T^, but was not closely related to any of the reference strains in the dendrogram.

Based on the data provided, strain 17EGHSPOS18-1^T^ represents a novel species in the genus *Anaerostipes*, for which the name *Anaerostipes stercorihominis* sp. nov. was proposed. The type strain is 17EGHSPOS18-1^T^ (=JCM 38261^T^ =DSM 121506^T^). Furthermore, as described in the Genome Features section, *A. hominis* and *A. faecis* share ANI and dDDH values that exceed the species discrimination thresholds, indicating that they should be assigned to the same taxon. These two species were validly published in 2021 and 2022, respectively, without comparison [8, 30, 31]. Based on these data, we propose *A. faecis* as a later heterotypic synonym of *A. hominis*.

## Description of *Anaerostipes stercorihominis* sp. nov.

*Anaerostipes stercorihominis* (ster.co.ri.ho’mi.nis. L. neut. n. *stercus*, dung; L. masc. n. *homo*, human being; N.L. gen. n. *stercorihominis*, of human faeces).

Cells are Gram-stain-positive in young cells but Gram-stain-variable in aged cells (>72 h). Spore formation is not seen from cells cultured in mGAM broth or BHIS broth. Catalase-negative straight rods (0.5×2.0–5.0 µm) and occur singly or in pairs. Growth is observed under anaerobic conditions. Colonies are white and ~2–3 mm in diameter under anaerobic conditions, but colonies are not developed under aerobic or microaerophilic (oxygen conc. 6–12%) conditions. Growth occurs at 30–45 °C (optimum at 37 °C) and at pH 6.0–8.0 (optimum at 7.0). Growth is observed in the broth containing 0–2% (wt/v) NaCl. Acid is produced from d-glucose, d-fructose, *myo*-inositol, mannitol, d-mannose, d-ribose and weakly produced from maltose and sucrose. Acid is not produced from cellobiose, d-fucose, d-galactose, d-gluconate, lactose or raffinose. Produce butyrate as a major end product with trace lactate and formate from d-glucose. Growth is enhanced in the presence of lactate as a sole carbon source. Positive enzyme reactions are obtained for acid phosphatase using API ZYM system, and weakly positive for alkaline phosphatase and naphthol-AS-BI-phosphohydrolase. Negative reactions recorded for *N*-acetyl-β-glucosaminidase, α-chymotrypsin, esterase, esterase lipase, α-fucosidase, α-galactosidase, α-glucosidase, β-galactosidase, β-glucosidase, β-glucuronidase, lipase, α-mannosidase, trypsin, cystine arylamidase, leucine arylamidase and valine arylamidase. The major cellular fatty acids are C_12:0_, C_18:0_ aldehyde and C_18 : 0_ DMA.

The type strain, 17EGHSPOS18-1^T^ (=JCM 38261^T^ =DSM 121506^T^), was isolated from a faeces of healthy adult man. The DNA G+C content of the type strain is 40.5 mol%. The GenBank/EMBL/DDBJ accession number for the 16S rRNA gene and the whole-genome sequence of this strain are LC897337 and BAAJCL000000000, respectively.

## Supplementary material

10.1099/ijsem.0.007276 Supplementary Material 1.

## Abbreviations

ANI: average nucleotide identity
ATCC: American Type Culture Collection
BHI: brain heart infusion
BHIS: brain heart infusion supplemented with yeast extract
dDDH: digital DNA–DNA hybridization
DMA: dimethyl acetal
GH: glycoside hydrolase
JCM: Japan Collection of Microorganisms
KEGG: Korean Encylopedia of Genes and Genomes
MALDI-TOF MS: matrix-assisted laser desorption/ionization time-of-flight mass spectrometry
mGAM: modified Gifu anaerobic medium
ML: maximum-likelihood
NBRC: NITE Biological Resource Center
NCBI: National Center for Biotechnology Information
SCFA: short chain fatty acid
UBCG2: Up-to-date-Bacterial Core Genes version 2
YCFA: yeast casitone fatty acid
