# Supplementary material for: Anaerostipes stercorihominis sp. nov., isolated from human faeces
Source: Int J Syst Evol Microbiol. 2026 Sep 7;76(9):007276. doi: 10.1099/ijsem.0.007276 (PMC13549443; doi:10.1099/ijsem.0.007276)
Supplement: Supplementary file 1 [file ijsem-76-07276-s001.pdf]

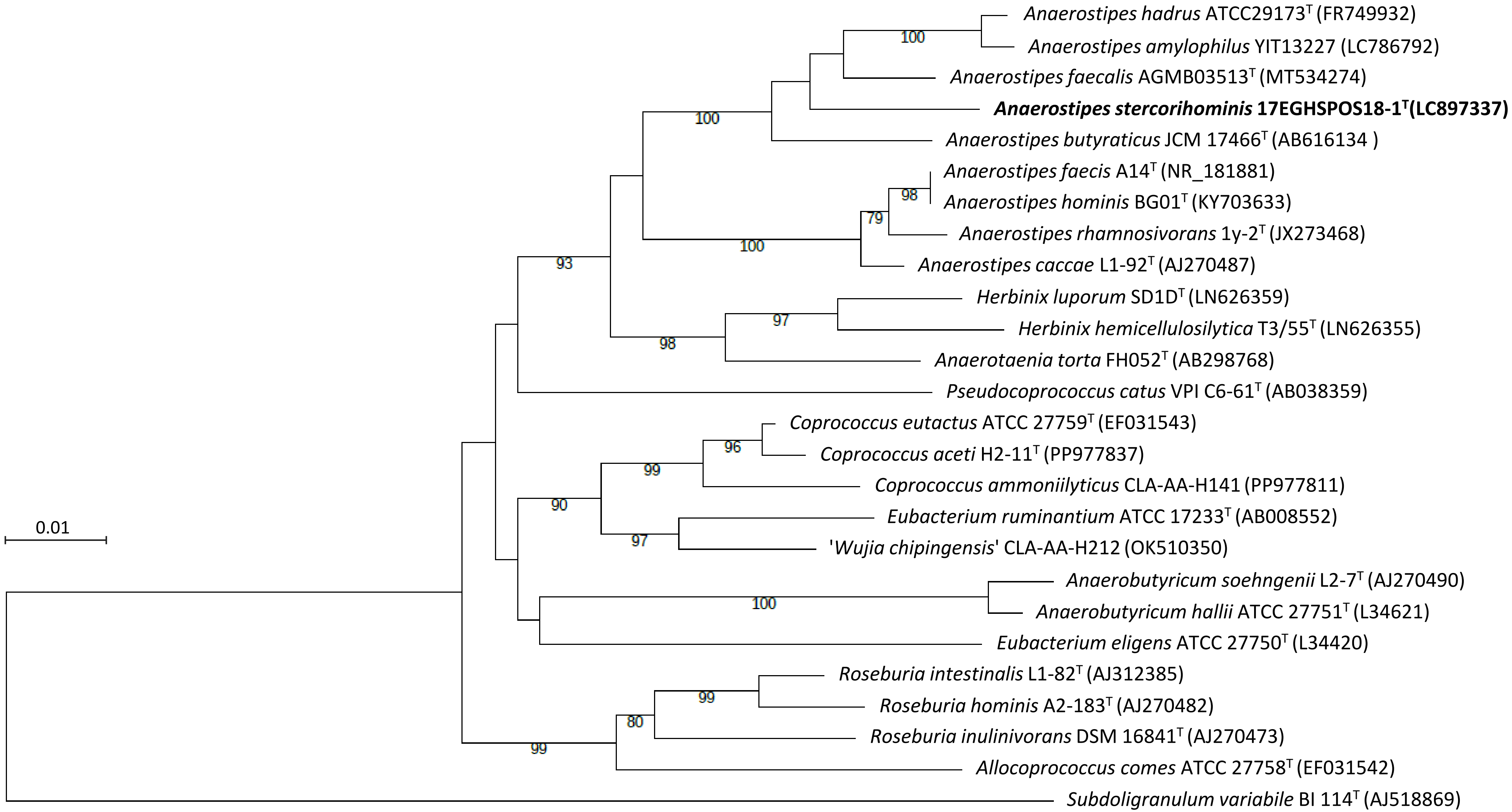

supplemental Fig. S1. ML phylogenetic tree based on the 16S rRNA gene sequences, showing the relationship between *A. stercorihominis* sp. nov. and the major gut butyrate producing bacteria in the family *Lachnospiraceae*. Bootstrap values (>70%) based on 100 replicates are shown at branch nodes. *Subdoligranulum variabile* BI 114^{T} was used as the outgroup. Bar shows 0.01 substitutions per nucleotide positions.

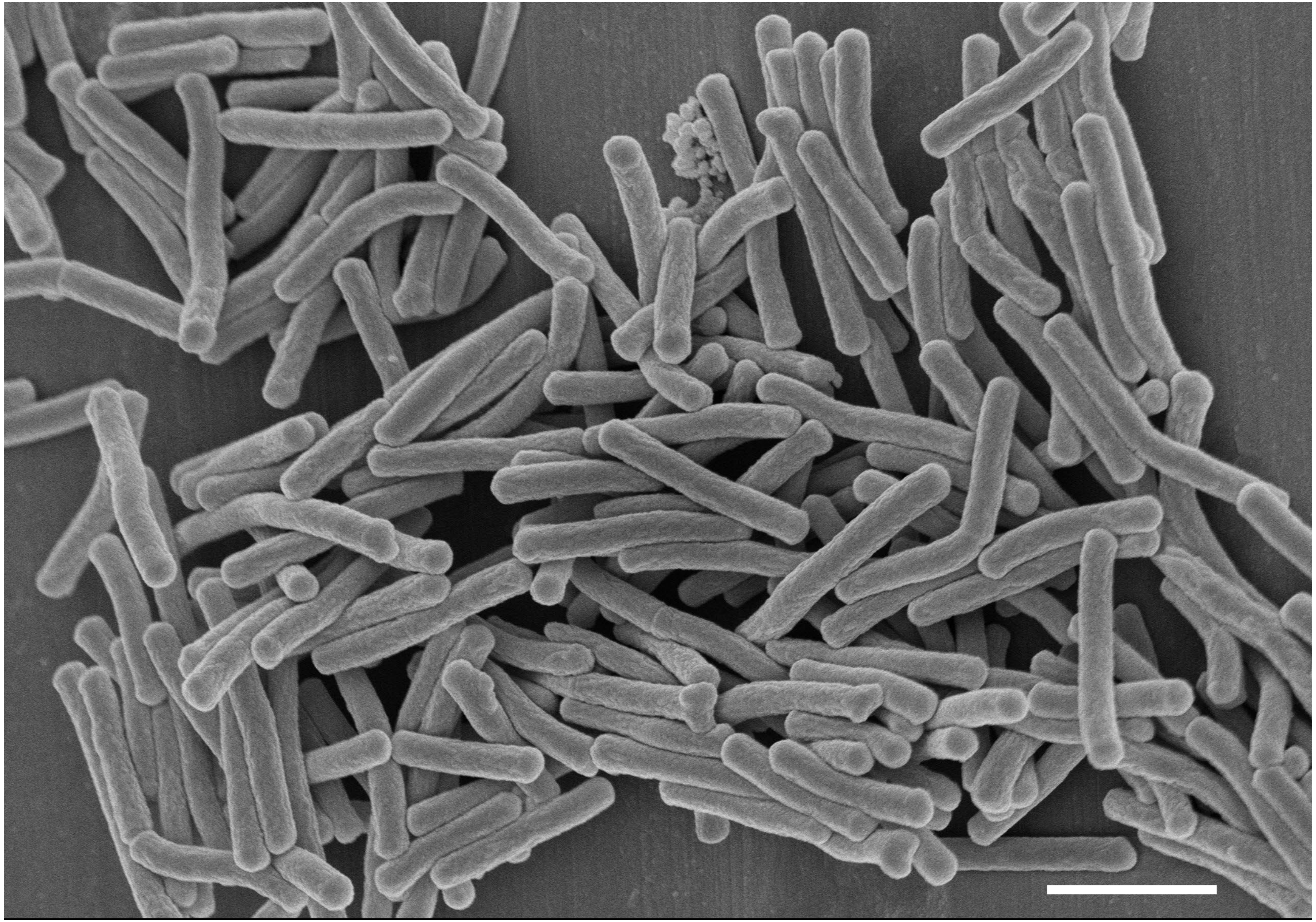

supplemental Fig. S2. Scanning electron micrograph of *A. stercorihominis* 17EGHSPOS18-1^{T}. Bar, 1 μm.

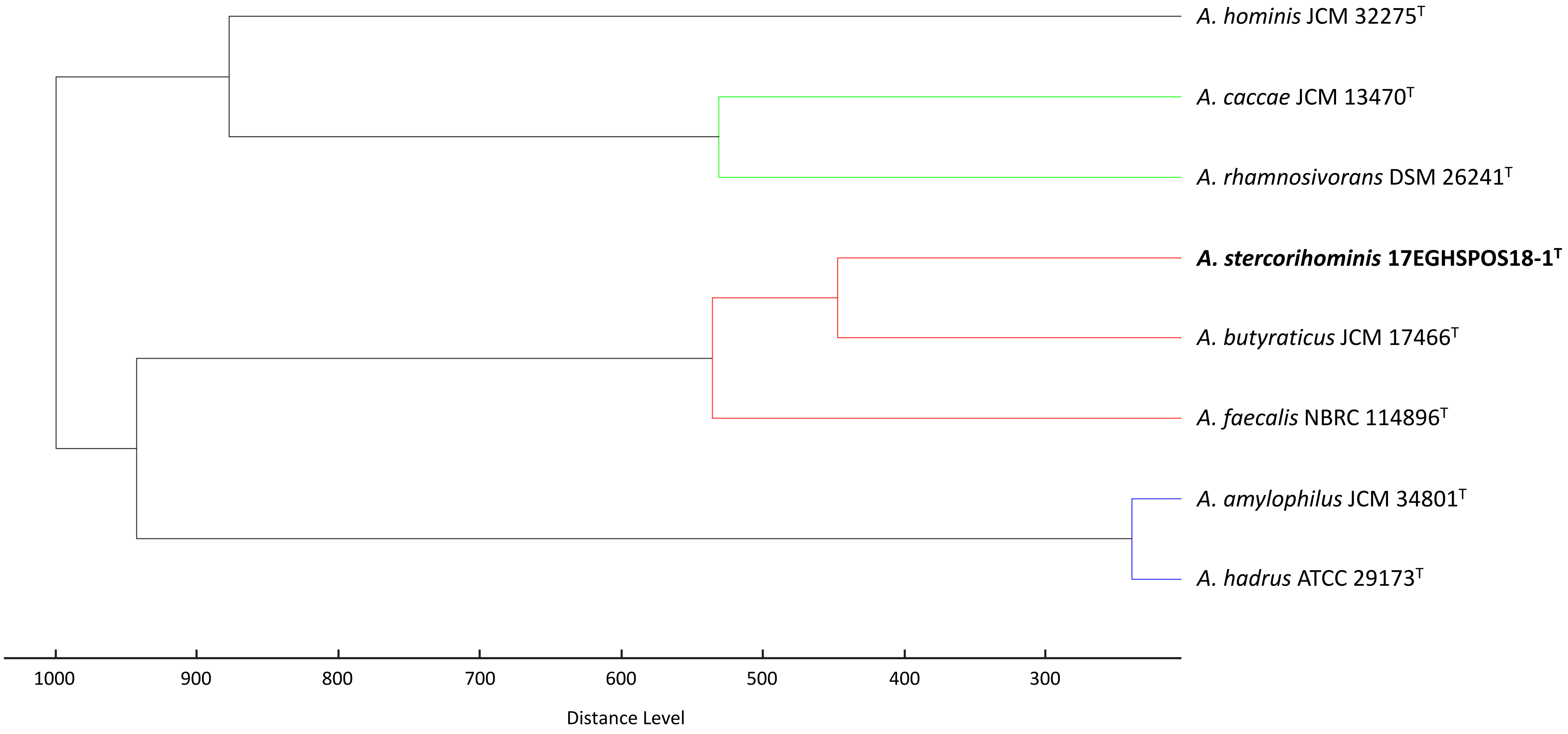

supplemental Fig. S3. MSP dendrogram generated with Biotyper software based on the spectra patterns of *Anaerostipes* spp.

supplemental Table S1. 16S rRNA gene sequence similarities among *Anaerostipes* spp.

			1	2	3	4	5	6	7	8	9
1	***A. stercorihominis* sp. nov.**	17EGHSPOS18-1^{T}	-	94.9	95.0	94.3	95.7	93.7	94.0	93.5	94.0
2	*A. hadrus*	DSM 3319^{T}		-	99.2	94.7	92.1	93.4	93.6	94.0	95.8
3	*A. amylophilus*	H1_26^{T}			-	94.8	95.9	93.3	93.4	93.9	92.1
4	*A. butyraticus*	JCM 17466^{T}				-	95.8	93.5	93.0	93.6	92.3
5	*A. faecalis*	AGMB03513^{T}					-	93.6	93.5	93.1	93.0
6	*A. caccae*	JCM 13470^{T}						-	97.2	96.9	98.0
7	*A. hominis*	BG01^{T}							-	97.4	99.6
8	*A. rhamnosivorans*	1y-2^{T}								-	93.2
9	*A. faecis*	A14^{T}									-

supplemental Table S2. Genomic features of the strains used in the present *in silico* study

					Number of			
Species	Strain	ID/assembly	Genome size (Mb)	G+C content (mol%)	contigs	Genes	CDSs	RNA
***A. stercorihominis* sp. nov.**	17EGHSPOS18-1[T]	GCF_057535655.1	2.7	40.5	59	2,732	2,611	59
A. hadrus	DSM 3319[T]	GCF_000332875.2	2.8	37.0	101	2,745	2,640	86
A. amylophilus	H1_26[T]	GCF_025567365.1	2.5	36.5	26	2,448	2,320	71
A. butyraticus	JCM 17466[T]	GCF_016586355.1	3.2	43.0	85	3,288	3,154	82
A. faecalis	AGMB03513[T]	GCF_018982945.1	2.5	37.0	37	2,598	2,489	67
A. caccae	JCM 13470[T]	GCF_014467075.1	3.6	44.5	1	3,525	3,427	69
A. hominis	BG01[T]	GCA_002270485.1	3.3	43.5	86	3,517	3,324	62
A. rhamnosivorans	1y-2[T]	GCF_005280655.1	3.6	44.5	1	3,558	3,454	70
A. faecis	A14[T]	GCF_910574135.1	3.2	44.0	63	3,377	3,179	76

supplemental Table S3. Cellular fatty acid compositions of *Anaerostipes* spp. Strains: 1, *A. stercorihominis* 17EGHSPOS18-1[T] (=JCM 38261[T]); 2, *A. hadrus* ATCC 29173[T]; 3, *A. amylophilus* JCM 34801[T]; 4, *A. butyraticus* JCM 17466[T]; 5, *A. faecalis*

Fatty acid	1	2	3	4	5
$C_{10:0}$	0.5	1.3	1.3	0.4	1.7
$C_{11:0}$	-	-	-	-	0.1
$C_{12:0}$	**36.0**	**56.6**	**26.4**	**47.5**	**23.1**
$C_{13:0}$	-	-	-	0.1	0.1
$C_{14:0}$	1.7	3.2	2.1	7.4	5.2
$C_{16:0}$	0.5	1.4	0.7	3.0	2.8
$C_{18:0}$	0.8	0.7	1.8	3.6	1.3
$C_{19:0}$	0.2	-	0.5	-	-
$C_{20:0}$	0.1	-	0.5	-	-
$C_{16:0}$ aldehyde	1.7	1.8	1.5	2.4	4.0
$C_{18:0}$ aldehyde	**17.4**	6.0	**16.4**	6.0	9.5
$C_{17:0}$ cyclo	0.3	-	0.2	-	0.2
iso-$C_{12:0}$	0.1	0.2	-	0.3	-
iso-$C_{13:0}$	0.3	0.6	0.1	2.5	0.1
anteiso-$C_{13:0}$	-	-	-	0.1	-
$C_{11:0}$ DMA	2.8	**13.6**	**10.8**	4.1	1.6
$C_{14:0}$ DMA	0.4	0.8	1.5	2.2	1.6
$C_{16:0}$ DMA	3.7	2.8	3.2	5.7	8.7
$C_{17:0}$ DMA	0.1	-	0.1	-	0.1
$C_{18:0}$ DMA	**32.4**	**10.6**	**31.3**	**12.8**	**17.7**
$C_{16:1}$ω9c	-	-	-	-	0.2
$C_{16:1}$ω7c	0.1	-	0.1	0.1	0.1
$C_{18:1}$ω9c	0.1	-	0.1	-	0.2
$C_{16:1}$ω7c DMA	0.1	-	0.1	0.1	0.5
$C_{18:1}$ω9c DMA	0.1	-	0.1	0.4	**10.4**
$C_{18:1}$ω7c DMA	0.2	-	0.1	0.1	3.0
$C_{18:1}$ AT 17.254 DMA	0.1	-	0.1	-	0.1
Summed Feature 1*	0.2	0.6	0.6	1.2	0.5
Summed Feature 2*	-	-	0.1	-	0.1
Summed Feature 4*	-	-	-	-	0.3
Summed Feature 5*	-	-	-	-	-
Summed Feature 6*	-	-	-	-	0.3
Summed Feature 7*	-	-	0.1	-	5.0
Summed Feature 8*	0.1	-	-	-	1.4
Summed Feature 10*	-	-	-	-	0.1

*Summed features are fatty acids that cannot be resolved reliably from another fatty acid using the chromatographic conditions chosen. The midi system groups these fatty acids together as one feature with a single percentage of the total. Summed feature compositions: 1, $C_{13:1}$ ω1c and/or $C_{14:0}$ aldehyde; 2, $C_{12:1}$ 3OH and/or $C_{13:0}$ DMA; 4, unknown 14.762 and/or $C_{15:2}$; 5, $C_{15:0}$ DMA and/or $C_{14:0}$ 3OH; 6, anteiso- $C_{15:0}$ 3OH and/or $C_{16:1}$ ω9c DMA; 7, $C_{17:2}$ and/or $C_{17:1}$ ω9c; 8, $C_{17:1}$ ω8c and/or $C_{17:2}$; 10, $C_{18:1}$ c11/t9/t6 and/or unknown 17.834. DMA, dimethyl acetal.
